# Nucleotide Discrimination with DNA Immobilized in the MspA Nanopore

**DOI:** 10.1371/journal.pone.0025723

**Published:** 2011-10-04

**Authors:** Elizabeth A. Manrao, Ian M. Derrington, Mikhail Pavlenok, Michael Niederweis, Jens H. Gundlach

**Affiliations:** 1 Department of Physics, University of Washington, Seattle, Washington, United States of America; 2 Department of Microbiology, University of Alabama at Birmingham, Birmingham, Alabama, United States of America; Université d'Evry val d'Essonne, France

## Abstract

Nanopore sequencing has the potential to become a fast and low-cost DNA sequencing platform. An ionic current passing through a small pore would directly map the sequence of single stranded DNA (ssDNA) driven through the constriction. The pore protein, MspA, derived from *Mycobacterium smegmatis*, has a short and narrow channel constriction ideally suited for nanopore sequencing. To study MspA's ability to resolve nucleotides, we held ssDNA within the pore using a biotin-NeutrAvidin complex. We show that homopolymers of adenine, cytosine, thymine, and guanine in MspA exhibit much larger current differences than in α-hemolysin. Additionally, methylated cytosine is distinguishable from unmethylated cytosine. We establish that single nucleotide substitutions within homopolymer ssDNA can be detected when held in MspA's constriction. Using genomic single nucleotide polymorphisms, we demonstrate that single nucleotides within random DNA can be identified. Our results indicate that MspA has high signal-to-noise ratio and the single nucleotide sensitivity desired for nanopore sequencing devices.

## Introduction

The ability to inexpensively sequence the genome of individuals would revolutionize both health care and the life sciences [1], [2] allowing for personalized medical treatments and better understanding of human traits and diseases [3], [4]. Furthermore, recognizing methylation sites within a sequence can contribute to the understanding of gene expression, human development and disease [5]–[7]. Although sequencing technologies have greatly improved in the last decade, most techniques still require the use of enzymes, chemicals, fluorescence and other expensive and resource intensive processes [8], [9]. In order to provide widespread DNA sequencing the cost must be further reduced.

Nanopores hold promise for inexpensive, fast, and nearly “reagent-free” DNA sequencing [10]. In its most basic form, an external voltage is applied across a nanometer-scale, electrolyte filled pore inducing an electric field that draws single stranded DNA (ssDNA) molecules into the constriction, modulating the ionic current through the pore. If each base-type, adenine (dA), cytosine (dC), thymine (dT), and guanine (dG), produces a unique residual current level, the time record of the ionic current can be used to identify nucleotides as they pass through the channel. It is the hope that non-standard bases, such as methylations, will also produce unique current signatures that can be read as part of the sequence.

Nanopore sequencing was first introduced using biological pore α-hemolysin (α-HL) [11], which continues to be the most commonly used channel. Homopolymeric strands produced distinct ionic currents and translocation times through the pore [11]–[15]. However, translocation times were very fast (1–7 µs/base) [11], [12] making it impossible to distinguish individual nucleotide signals from the noisy background [16]. The necessary reduction of DNA translocation speed may be achieved by using modified DNA [17] or by using unmodified DNA but involving a molecular motor [18]–[20]. Such methods slow or halt the strand within the pore long enough to obtain sufficient signal-to-noise for individual nucleotides.

In this paper we explore the ion current levels and nucleotide resolution expected in strand sequencing of unmodified (native) DNA using a technique similar to that first used with α-HL pores. When ssDNA was immobilized inα-HL by attachment to streptavidin, a molecule too large to pass through the pore, homopolymer strands of adenine, cytosine, and thymine each produced a distinct, orientation dependent, current blockade [13], single nucleotide differences in heteromeric DNA could be resolved [14], [15], and methylated cytosine could be distinguished from unmethylated cytosine [21]. However, because of α-HL's long β-barrel, the current differences are small. Furthermore, α-HL has several recognition sites that contribute to the ionic current [14], [15] making single nucleotide resolution challenging.

As an alternative to α-HL, our group introduced MspA, a biological pore derived from *Mycobacterium smegmatis*, to nanopore sequencing. MspA has a single short (∼0.5 nm long) and narrow (∼1 nm in diameter) constriction [22], [23]. Since negative charges near the constriction of wild type MspA prohibited DNA translocation, we developed a mutant, denoted M1-MspA, in which three negatively charged aspartic acids were replaced with neutral asparagines, resulting in a neutral constriction [23]. When DNA was immobilized in M1-MspA using a duplex region, the four standard homopolymers caused distinct ionic currents and single nucleotides in a homopolymer background were resolved [17]. However, the residual current levels in free streaming or enzyme controlled DNA translocations [18]–[20] are expected to be different from the current levels of DNA held by duplex sections since the duplex region also affects the ionic current [17].

**Figure 1:**
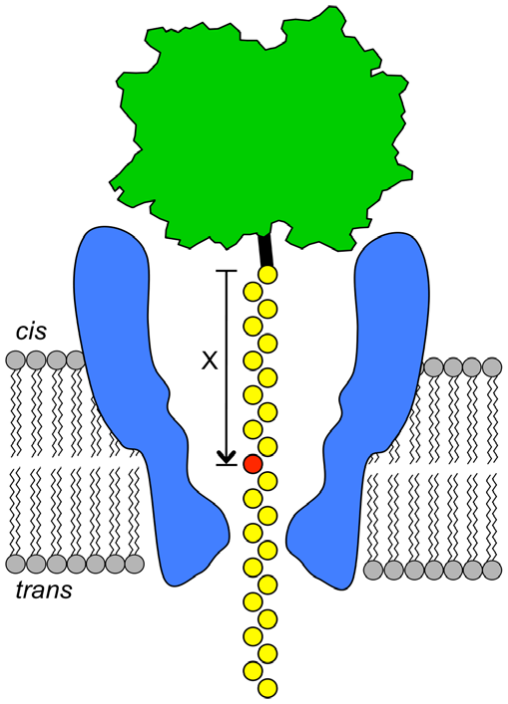
Schematic diagram. Schematic diagram of MspA (blue) set up in a lipid bilayer (grey). Single stranded DNA (ssDNA) was attached to a bulky NeutrAvidin molecule (green) using a biotin linker (black). A specific nucleotide (red) is designated by it's position, X, from the biotin-NeutrAvidin ‘anchor’. The ssDNA threads through the pore from the *cis* side of the bilayer until the bulky NeutrAvidin prevents it from further translocation. Residual ion current was recorded as the ssDNA is immobilized within MspA.

In this work, NeutrAvidin was used to hold ssDNA in the constriction of MspA in a similar manner as a processive enzyme would hold ssDNA, allowing long duration current readings (See Figure 1). Using these NeutrAvidin anchored ssDNAs, we established the nucleotide resolving power of M1-MspA by altering the nucleotides positioned in or near the constriction and recording the characteristic current. Although the NeutrAvidin molecule is bulky, it does not appreciably affect the measured ionic current [14]. First, we determined residual current levels of each base type by using homopolymeric strands and established the current level for methylated cytosine (mC). Next, we characterized the recognition site within the pore using single nucleotide substitutions in an otherwise homopolymeric ssDNA. Finally, using segments of genomic sequences containing a single nucleotide polymorphism (SNP), we demonstrated that the ionic current through M1-MspA could be used to differentiate single nucleotide substitutions in heteromeric ssDNA.

## Results

### Nucleotide Current Signatures

When a voltage was applied across a lipid bilayer containing an M1-MspA porin, the ssDNA of a ssDNA-NeutrAvidin complex could thread through M1-MspA, reducing the current through the pore to a nucleotide-dependent level of about 25% of the unblocked value (see Figure S1 for an example event). The ssDNA remained threaded through the pore until it was ejected back into to the *cis* volume by reversing the voltage. We calculated average residual current levels when the ssDNA was immobilized in M1-MspA under an applied voltage of 180 mV. The current distributions for each type of ssDNA were highly repeatable on several different M1-MspA pores (n>3) (see Table S1).

**Figure 2:**
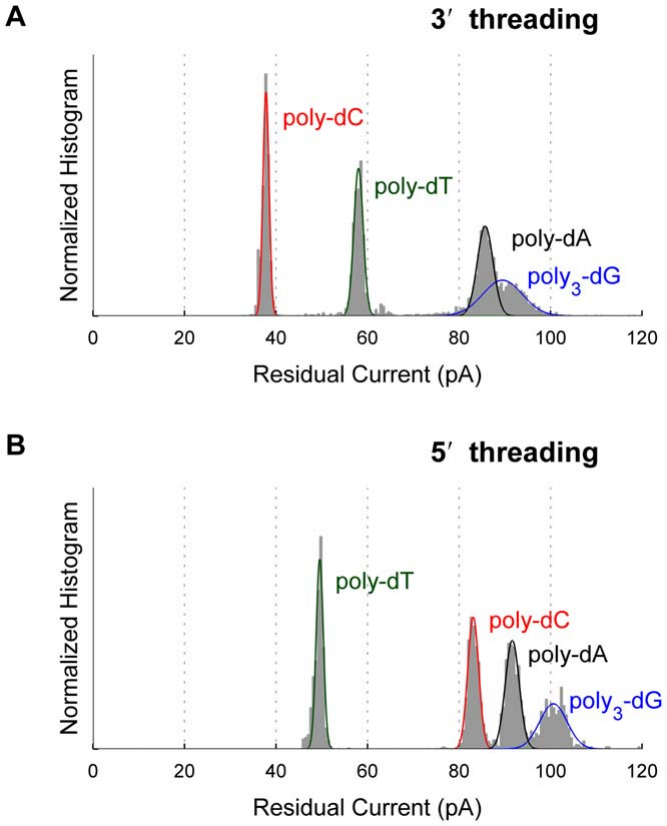
Orientation specific homopolymer histograms. Mean residual ionic current (gray) and fitted Gaussian curves are shown for homopolymer adenine (‘poly-dA’, black), cytosine (‘poly-dC’, red), thymine (‘poly-dT’, green), and guanine (‘poly_3_-dG’, blue) for (A) 3′ threading and (B) 5′ threading. With 3′ threading, poly-dA and poly_3_-dG overlap by 27% of the area of the two Gaussians. With 5′ threading, poly-dA and poly_3_-dG overlap by 2% and poly-dC and poly-dA overlap by 0.1% of the area of the two Gaussians.

We recorded residual currents for ssDNA of homopolymer cytosine, (dC)_50_ (denoted ‘poly-dC’), adenine (dA)_50_ (‘poly-dA’), and thymine (dT)_50_ (‘poly-dT’). Although we could not study homopolymer guanine due to G-tetrad formation, we examined 3 guanines in a homopolymer adenine background, (dA)_13_(dG)_3_(dA)_34_, (denoted ‘poly_3_-dG’). The NeutrAvidin molecule was bound to these homopolymers by a biotin linker attached to either the 5′ or 3′ end so that the 3′or 5′end, respectively, threaded through the pore (see Figure 1). Histograms of the mean residual current and fitted Gaussians of each homopolymer for the 3′ and 5′ threading orientations are shown in Figure 2. The residual current levels are highly sensitive to the identity and orientation of nucleotides within M1-MspA. For both 3′ and 5′ threading, the residual currents for poly-dA, poly-dC, and poly-dT are well resolved with current separations of at least ∼8 pA (0.04 nS) and up to ∼50 pA (0.28 nS). For 3′ threading, poly-dA and poly_3_-dG overlap by 27% of the area of the two Gaussian distributions. For 5′ threading, the residual currents for poly-dC, poly-dA, and poly_3_-dG are well resolved with minimal overlap and the current of poly-dT is separated from the other current levels by at least 33 pA (0.18 nS). For poly-dC we find a substantial orientation dependence with a difference in the current of 5′ vs. 3′ threading of 45 pA (0.25 nS). For both orientations, the histograms of residual current for poly_3_-dG are wider than those of the other homopolymeric strands. This breadth is possibly due to the surrounding adenine nucleotides influencing the ionic current. Neglecting poly_3_-dG, there is a larger current distinction for 3′ threaded homopolymers. Therefore, we used 3′ threading for the remainder of our experiments described here.

**Figure 3:**
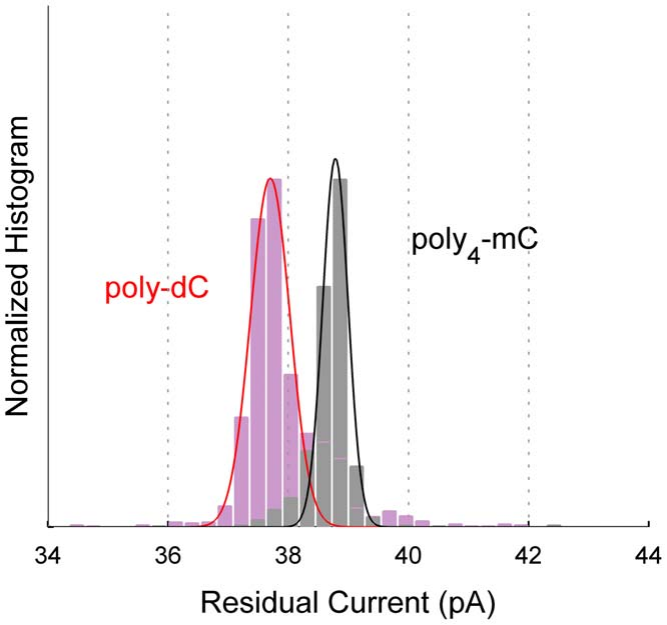
Methylated cytosine. Mean residual ionic currents and the fitted Gaussian curve are shown for ssDNA of homopolymer cytosine (‘poly-dC’, red) and a strand with 4 methylated cytosines (‘poly_4_-mC’, black) substituted at the 13^th^–16^th^ nucleotides from the NeutrAvidin anchor in an otherwise homopolymer cytosine strand. Experiments were conducted on three pores with poly_4_-mC and poly-dC added sequentially to each pore. The peaks of the Gaussians are separated by 1.1 pA and the curves overlap by ∼2% of the area of the two Gaussians.

To determine whether M1-MspA can resolve methylated cytosine, we compared residual current levels of 5′/biotin/(dC)_12_(mC)_4_(dC)_14_ 3′, (abbreviated ‘poly_4_-mC’), and poly-dC. To ensure proper calibration between experiments, poly_4_-mC and poly-dC were added sequentially to each pore. The peak current levels for poly-dC and poly_4_-mC are separated by ∼1.1 pA (0.006 nS) with an overlap of ∼2% of the area of the two Gaussians (see Figure 3).

### Region of Sensitivity

To realize nanopore DNA sequencing, the residual current of each nucleotide passing though the pore must be identifiable. This requires a short region over which the pore recognizes nucleotides. Armed with the residual current signatures of each base type, we mapped the region within the anchored ssDNA that is most influential in determining the ionic current through M1-MspA. Based on the crystal structure of MspA [22], approximately the first 20 nucleotides reside within its lumen or the constriction when the NeutrAvidin molecule is positioned above the rim of MspA. It was expected that longer DNA strands would reach through the constriction of the pore to the *trans* side. We used ssDNA comprised of distinct regions of homopolymeric cytosine, adenine, and thymine to narrow in on the region of nucleotide sensitivity. By comparing the residual ionic current of these strands to the current signatures found above for the homopolymeric strands, the nucleotides most affecting the ionic current were determined to be around the 13th to 15th nucleotides from the NeutrAvidin (see Figure S2).

### Single Nucleotide Substitutions in a Homopolymeric Background

**Figure 4:**
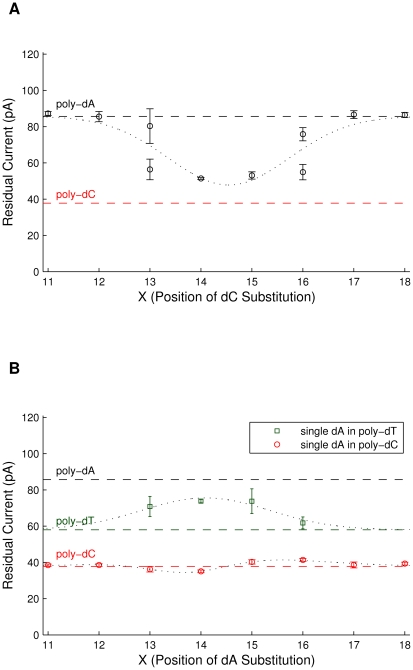
Single heteromeric substitutions in homopolymer ssDNA. Peak values and half width half height values, represented as error bars, of fitted Gaussians of mean residual ionic currents are shown for experiments with a single heteromeric substitution in homopolymer ssDNA. For comparison, the mean residual ionic currents for relevant homopolymer strands are shown as dashed lines. Gaussian curves are included to aid the eye. (A) DNA strands containing a single cytosine (dC) substituted at nucleotide position X from the NeutrAvidin anchor in an otherwise poly-dA strand. Large error bars (X = 13) indicate a wide distribution and the two points at X = 13 and X = 16 indicate two current levels for that substitution. The residual current differs most from that of poly-dA (black dashed line), most resembling that of poly-dC (red dashed line) at X = 14 and 15. (B) DNA strands containing a single cytosine (dA) substituted at nucleotide position X from the NeutrAvidin anchor in an otherwise poly-dC (red) or poly-dT (green) strand. A single dA substitution in homopolymer poly-dC yields a residual current similar to that of poly-dC (red dashed). For a single dA substitution in homopolymer poly-dT, the current deviates most from that of poly-dT (green dashed) towards the level for poly-dA (black-dashed) at X = 14 and 15.

To determine which individual nucleotides most affect the ionic current, we introduced a single cytosine, dC, in a homopolymer adenine strand at the 11th through 18th nucleotide from the NeutrAvidin anchor (denoted dC_X_ where X is the position of substitution). Figure 4a shows a plot of the residual ionic currents for each position of dC substitution. The residual current deviates most from that of poly-dA for dC_14_ and dC_15_, reducing the current to a level intermediate to that of poly-dC and poly-dA. For dC_13_ as well as for dC_16_, the histograms of the current distributions have two separate peaks that are wider than those for dC_14_ and dC_15_. The increased widths of the current distribution seen for dC_13_ and dC_16_ and the for above-described poly_3_-dG, where three guanine nucleotides are surrounded by adenines, are consistent with the nucleotides of interest shifting in and out of the constriction, possibly due to thermal fluctuations, transient bindings and variations in the seating of the NeutrAvidin anchor on the rim of M1-MspA. With these data, we conclude that there is a single region in M1-MspA that is sensitive to nucleotides. Assuming a Gaussian distribution, the sensitive region corresponds to X = 14.5 with a half width at half height of 1.5 nucleotides. This indicates that the nucleotides centered in MspA's constriction as well as the neighboring nucleotides just outside the constriction affect the ionic current.

To confirm the location of the sensitive region, we similarly examined a single adenine substitution, dA_X_ in poly-dC (see Figure 4b). The dA_X_ substitutions in poly-dC influence ionic current much less than the dC_X_ substitutions in poly-dA found above. To determine whether this effect is the influence of the homopolymer background on the ionic current, we also studied four different dA_X_ substitutions in an otherwise poly-dT strand (see Figure 4b). We see that the current levels for dA_X_ substitutions in poly-dT are distinct from poly-dT at X = 13, 14, 15, and 16. A fitted Gaussian gives a peak value at X = 14.1 and half width at half height of 1.5. In total, these results suggest that the ionic current is most influenced by the nucleotides at positions X = 14–15 and that the homopolymer background affects the ability of M1-MspA to recognize single nucleotides.

### Single Nucleotide Substitutions in a Heteropolymeric Background

**Figure 5:**
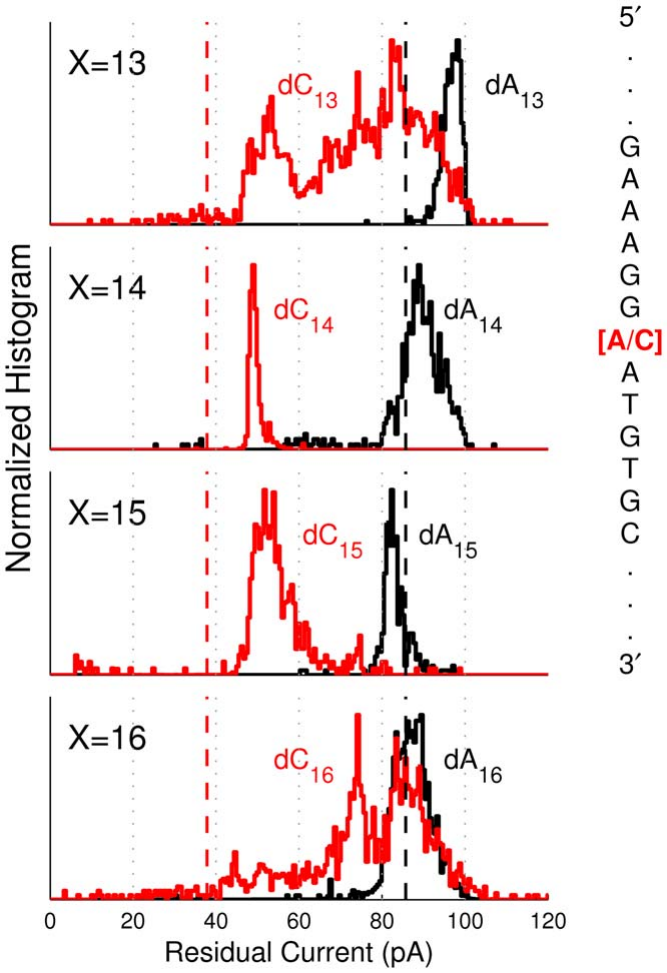
SNP histograms (rs889312). A segment of DNA containing SNP rs889312 was bound to NeutrAvidin such that the polymorphism is at the 13–16^th^ position from the NeutrAvidin anchor (denoted dC_X_ or dA_X_ where X was the position of the SNP). Part of the surrounding sequence is shown with the SNP highlighted in red (see Table S1 for complete sequence). Histograms of mean residual current levels are shown for each variant and SNP location, X = 13–16. The two variations are most clearly resolved for X = 14 and 15. For reference, the mean residual ionic currents for poly-dC (red dashed) and poly-dA (black dashed) are shown.

To investigate the prospect of M1-MspA to identify a single nucleotide in a heteromeric background, we prepared genomic sequence DNA with single nucleotide substitutions. Specifically, we analyzed DNA containing the single nucleotide polymorphism (SNP) rs889312, which has been linked to increased risk of breast cancer [24]. The ssDNA was bound to NeutrAvidin such that the SNP (either dC or dA) was at X = 13, 14, 15 or 16 (denoted dC_X_ or dA_X_ where X is the position of the SNP). Histograms for the residual ionic currents for each SNP substitution are shown in Figure 5. The dA and dC variations are clearly distinguishable for X = 14 and 15 and become complex and poorly resolved for X = 13 and 16. This procedure was repeated for a second SNP, rs1447295, which is associated with an increased risk of prostate cancer [25], [26]. For rs1447295 we also observe two highly resolved current levels distinguishing the two SNP variants (see Figure S3). For both SNP experiments, M1-MspA was able to clearly distinguish single nucleotide differences in genomic DNA in its sensitive region.

## Discussion

The differences in nucleotide specific current found with M1-MspA are about an order of magnitude larger than those found in similar experiments with α-HL [13]–[15]. Additionally, M1-MspA has a single recognition site where nucleotides affect the ionic current whereas α-HL has several such regions [14], [15]. The short M1-MspA constriction results in a small volume of high current density which is likely responsible for the large open state conductance and the large specificity to various nucleotides in the constriction. The ionic current for poly-dC is significantly higher for the 5′ threading orientation in M1-MspA. This is opposite to α-HL [13], suggesting an orientation dependent interaction with the porin. A simple resistor model wherein the total resistance is determined by the algebraic sum of the resistances posed by each nucleotide and the pore cross section at the position of that nucleotide [15] does not properly describe our data. Our results also do not support a rate-limiting model where the highest impedance in ionic current controls the measured current level [17]. It seems that a more complex model accounting for detailed positioning, hydration, charge distributions and binding affinity of nucleotides to the pore is needed to properly describe this system. Additionally, our data show that nanopore sequencing data analysis of DNA must take into account the effect of neighboring nucleotides not centered in the constriction.

Compared to our own data with DNA held by a duplex section in MspA [17] we find larger currents and current differences as well as a slight reordering of the nucleotide specific current levels. An increase in residual current when DNA is anchored with a large molecule is expected [14] and may be due to elongation and straightening of the DNA under the force provided by the electric field of the constriction. Additionally, in DNA held by a duplex region the length and sequence of a duplex region near the constriction throttles the current and affects the neighboring single stranded bases occupying the constriction [17].

We have shown that homopolymer ssDNA is well resolved in M1-MspA and methylated cytosine is distinguishable from unmethylated cytosine. M1-MspA is capable of high-resolution single nucleotide discrimination, a prerequisite for DNA nanopore sequencing. Future mutations of MspA utilizing transient binding to nucleotides may be used to slow ssDNA translocation as well as for tailoring the current levels. Additionally, a technique employing a DNA polymerase to ratchet ssDNA has been shown to move DNA, one base at a time, through the α-HL pore [18]–[20], [27]–[29]. Due to the similarity in geometry of the immobilization technique, we expect that the nucleotide specific current levels found here with DNA held by NeutrAvidin will be similar to those that will be found with polymerase activated DNA translocation through M1-MspA. The procession time of DNA polymerases such as the Klenow Fragment or phi29 is sufficiently slow, >20 ms/nt, that MspA would be able to resolve bases in order as they pass through the pore. In total, MspA's advantages for fast nanopore sequencing are apparent with its single short region of high sensitivity to nucleotide type and sharp resolution to nucleotide position. MspA will likely play an important role in the future of DNA nanopore sequencing.

## Materials and Methods

### Experiments

Pores were established with previously described methods [23]. Briefly, lipid bilayers were formed across a horizontal ∼20 µm diameter aperture in Teflon from 1,2-diphytanoyl-sn-glycerol-3-phosphocholine, 1,2-diphytanoyl-sn-glycero-3-phosphate (Avanti Polar Lipids) or equal mixtures thereof. Compartments on both sides of the bilayer contained experimental buffer of 1.0 M KCl, 10 mM HEPES/KOH buffered at pH 8.0+/−0.05. An Axopatch-1B or 1C patch clamp amplifier (Axon Instruments) applied a voltage across the bilayer and measured the ionic current. M1-MspA was added to the grounded *cis* compartment at a concentration of ∼2.5 ng/ml. Once a single protein inserted, the compartment was flushed with experimental buffer in order to avoid further insertions. All experiments were performed at 23±1°C. The analog signal was low-pass filtered at 20 kHz with a 4-pole Bessel filter and then digitized at 100 kHz. Data acquisition was controlled with custom software written in LabWindows/CVI (National Instruments). The pore was held at 180 mV until there was a spontaneous reduction in current to less than 200 pA that lasted longer than 100 ms, signifying DNA immobilized within the pore. The amplifier applied 180, 160, 140, 120, 100, and 80 mV for 250 ms per voltage level then applied −100 mV to force the DNA out of the pore back into the *cis* compartment. The voltage then returned to 180 mV. See Figure S1 for an example event. Currents described herein correspond to an applied voltage of 180 mV; currents at lower voltages were not examined in this study.

### Biological Materials

The M1-MspA protein was purified from *Mycobacterium smegmatis* as previously described [23]. DNA with biotin on either the 5′ or 3′ end was synthesized by Integrated DNA Technologies with standard desalting and no additional purification. NeutrAvidin was obtained from Invitrogen. DNA was mixed in equal molar concentrations with NeutrAvidin to create ssDNA-NeutrAvidin complexes, which were stored at −20°C until immediately before use. For experiments, 5 µM of the ssDNA-NeutrAvidin complex was added to the *cis* chamber.

### Data Analysis

Data was analyzed with custom software written in Matlab (The Mathworks). Translocation of DNA was first identified using current thresholds as described in Butler [23]. Minor variations in open-pore current levels were seen across a number of experiments and are likely due to minor changes in buffer conditions influencing conductivity. Fluctuations between experiments were minimized by normalizing the residual current for each translocation by the unblocked current level for that experiment. Events with a standard deviation in current of more than twice the average deviation for the DNA strand type were discarded. To report values in current, we multiplied these normalized-currents by the average open-pore current for all experiments, 328+/−11 pA (mean +/− std dev), for an applied voltage of 180 mV. Subsequent experiments were often conducted with DNA strands of different compositions sequentially added without perfusing. After each new DNA addition, a corresponding peak appeared in the histogram of residual currents. For each DNA strand type, data was taken on several pores (see Table S1). The ordering of DNA additions was varied to provide at least one experiment in which the histogram peak was clearly resolved with no overlap from other strand types. The mean current values and errors reported herein were determined by the peak value and half width at half height of a Gaussian fitted to the histogram of mean residual currents for all experiments (see Table S1).

## Supporting Information

Figure S1 **Example data.** A current (upper) and voltage (lower) trace is shown for an example event. The applied voltage was held at 180 mV until there was a spontaneous reduction in current corresponding to DNA entering the pore. The voltage was held at 180, 160, 140, 120, 100 and 80 mV for 250 ms per voltage level. Then, −100 mV was applied to force the DNA out of the pore and back into the *cis* compartment. Residual current levels reported in this paper correspond to an applied voltage of 180 mV. The transient spikes seen when the voltage is held at 180 mV result from DNA entering the pore vestibule but escaping before fully threading through the constriction.
(TIF)

Figure S2 **Region of sensitivity.** To determine the region of sensitivity of M1-MspA, the residual ion current was measured when the two strands of ssDNA shown below were held within the pore with a NeutrAvidin ‘anchor’. The mean residual ionic current (gray) and fitted Gaussian curve (black) are shown for each strand. The mean residual ionic current for ‘Strand 1’ is most like that of poly-dC, suggesting a recognition site within the first 15 bases (red). The mean residual ionic current for ‘Strand 2’ is most like that of poly-dA, corresponding to a region of sensitivity near the 13^th^–15^th^ nucleotide (red). For reference, the Gaussian means of the mean residual ionic current are shown for poly-dC (red dashed), poly-dT (green dashed), and poly-dA (black dashed).
(TIF)

Figure S3 **SNP histograms (rs1447295).** A segment of ssDNA containing SNP rs1447295, which is associated with an increased risk of prostate cancer [25], [26], was bound to NeutrAvidin such that the polymorphism is at the 13–16^th^ nucleotide, X, from the NeutrAvidin. Part of the surrounding sequence is shown with the nucleotide of interest, either an adenine or cytosine, highlighted in red (see Table S1 for complete sequences). Histograms of the mean residual ionic current for both the dA_X_ and dC_X_ variants for X = 13–16 and Gaussian means of the mean residual ionic current for poly-dA (black dashed) and poly-dC (red dashed) are shown. The two variants are most resolved when X = 13 and 14. The ionic current level for dA_X_ is similar to that of poly-dA and the current for dC_X_ is similar to that found for a single dC in poly-dA. When X = 15 or 16 we observe two current levels for each variation; one level is near 40 pA for both dA_X_ and dC_X_ and the other level is unique to the SNP variation.
(TIF)

Table S1 **Supporting table.**
(XLS)

## References

[1] Bentley DR (2006). Whole-genome re-sequencing.. Curr Opin Genetics Dev.

[2] Kahvejian A, Quackenbush J, Thompson JF (2008). What would you do if you could sequence everything?. Nature Biotechnol.

[3] Hirschhorn JN (2009). Genomewide Association Studies – Illuminating Biologic Pathways.. N Engl J Med.

[4] Goldstein DB (2009). Common Genetic Variation and Human Traits.. N Engl J Med.

[5] Cokus SJ, Feng S, Zhang X, Chen Z, Merriman B (2008). Shotgun bisulphite sequencing of the Arabidopsis genome reveals DNA methylation patterning.. Nature.

[6] Robertson KD (2005). DNA methylation and human disease.. Nature Rev Genet.

[7] Razin A, Shemer R (1995). DNA methylation in early development.. Hum Mol Gen.

[8] Shendure J, Ji H (2008). Next-generation DNA sequencing.. Nature Biotechnol.

[9] Fuller CW, Middendorf LR, Benner SA, Church GM, Harris T (2009). The challenges of sequencing by synthesis.. Nature Biotechnol.

[10] Branton D, Deamer DW, Marziali A, Bayley H, Benner SA (2008). The potential and challenges of nanopore sequencing.. Nature Biotechnol.

[11] Kasianowicz JJ, Brandin E, Branton D, Deamer DW (1996). Characterization of individual polynucleotide molecules using a membrane channel.. Proc Natl Acad Sci USA.

[12] Meller A, Nivon L, Brandin E, Golovchenko J, Branton D (2000). Rapid nanopore discrimination between single polynucleotide molecules.. Proc Natl Acad Sci USA.

[13] Purnell RF, Mehta KK, Schmidt JJ (2008). Nucleotide identification and orientation discrimination of DNA Homopolymers immobilized in a protein nanopore.. Nano Lett.

[14] Stoddart D, Heron AJ, Mikhailova E, Maglia G, Bayley H (2009). Single-nucleotide discrimination in immobilized DNA oligonucleotides with a biological nanopore.. Proc Natl Acad Sci USA.

[15] Purnell RF, Schmidt JJ (2009). Discrimination of Single Base Substitutions in a DNA Strand Immobilized in a Biological Nanopore.. ACS Nano.

[16] Mitchell N, Howorka S (2008). Chemical tags facilitate the sensing of individual DNA strands with nanopores.. Angew Chem Int Ed.

[17] Derrington IM, Butler TZ, Collins MD, Manrao E, Pavlenok M (2010). Nanopore DNA sequencing with MspA.. Proc Natl Acad Sci USA.

[18] Cockroft SL, Chu J, Amorin M, Ghadiri MR (2008). A single-molecule nanopore device detects DNA polymerase activity with single-nucleotide resolution.. J Am Chem Soc.

[19] Lieberman KR, Cherf GM, Doody MJ, Olasagasti F, Kolodji Y (2010). Processive Replication of Single DNA Molecules in a Nanopore Catalyzed by phi29 DNA Polymerase.. J Am Chem Soc.

[20] Benner S, Chen RJA, Wilson NA, Abu-Shumays R, Hurt N (2007). Sequence-specific detection of individual DNA polymerase complexes in real time using a nanopore.. Nat Nanotechnol.

[21] Wallace EVB, Stoddart D, Heron AJ, Mikhailova E, Maglia G (2010). Identification of epigenetic DNA modifications with a protein nanopore.. Chem Commun.

[22] Faller M, Niederweis M, Schulz GE (2004). The structure of a mycobacterial outer-membrane channel.. Science.

[23] Butler TZ, Pavlenok M, Derrington IM, Niederweis M, Gundlach JH (2008). Single-molecule DNA detection with an engineered MspA protein nanopore.. Proc Natl Acad Sci USA.

[24] Easton DF, Pooley KA, Dunning AM, Pharoah PD, Thompson D (2007). Genome-wide association study identifies novel breast cancer susceptibility loci.. Nature.

[25] Amundadottir LT, Sulem P, Gudmundsson J, Helgason A, Baker A (2006). A common variant associated with prostate cancer in European and African populations.. Nat Genet.

[26] Freedman ML, Haiman CA, Patterson N, McDonald GJ, Tandon A (2006). Admixture mapping identifies 8q24 as a prostate cancer risk locus in African-American men.. Proc Natl Acad Sci USA.

[27] Gyarfas B, Olasagasti F, Benner S, Garalde D, Lieberman KR (2009). Mapping the Position of DNA Polymerase-Bound DNA Templates in a Nanopore at 5 angstrom Resolution.. ACS Nano.

[28] Wilson NA, Abu-Shumays R, Gyarfas B, Wang H, Lieberman KR (2009). Electronic control of DNA polymerase binding and unbinding to single DNA molecules.. ACS Nano.

[29] Olasagasti F, Lieberman KR, Benner S, Cherf GM, Dahl JM (2010). Replication of individual DNA molecules under electronic control using a protein nanopore.. Nature Nanotechnol.

