# Supplementary figures and images for: Nucleotide Discrimination with DNA Immobilized in the MspA Nanopore
Source: PLoS One. 2011 Oct 4;6(10):e25723. doi: 10.1371/journal.pone.0025723 (PMC3186796; doi:10.1371/journal.pone.0025723)

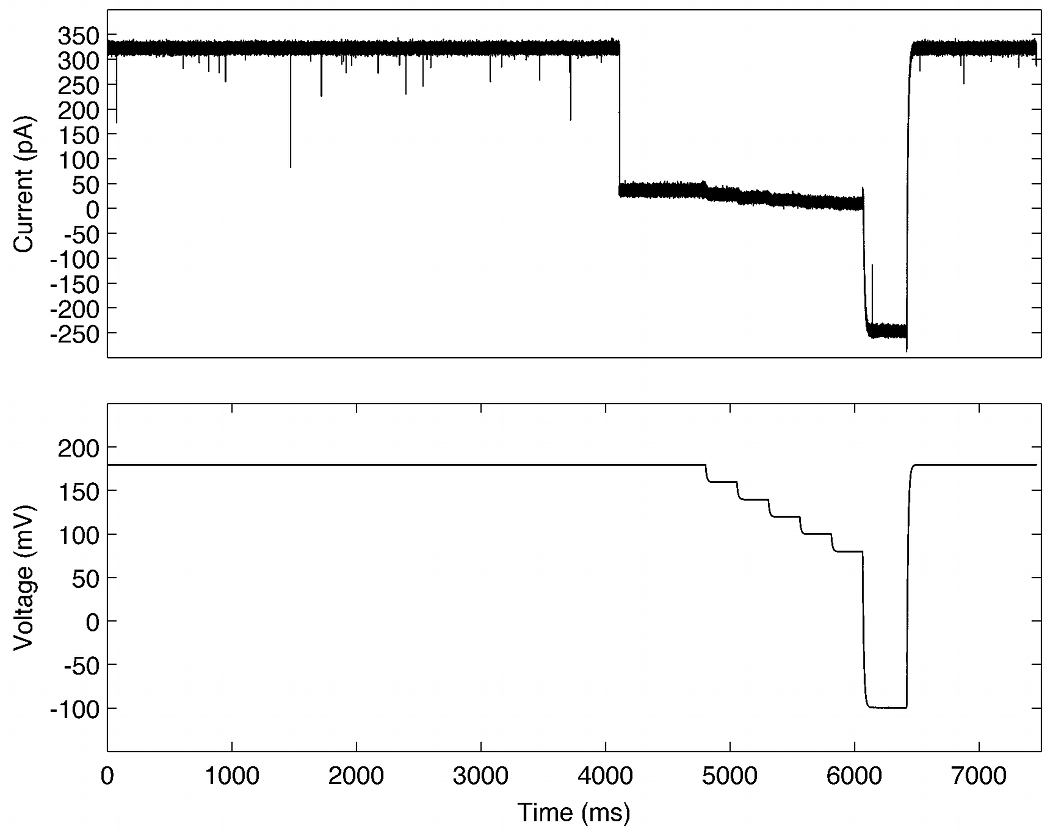

Supplement: Figure S1 — Example data. A current (upper) and voltage (lower) trace is shown for an example event. The applied voltage was held at 180 mV until there was a spontaneous reduction in current corresponding to DNA entering the pore. The voltage was held at 180, 160, 140, 120, 100 and 80 mV for 250 ms per voltage level. Then, −100 mV was applied to force the DNA out of the pore and back into the cis compartment. Residual current levels reported in this paper correspond to an applied voltage of 180 mV. The transient spikes seen when the voltage is held at 180 mV result from DNA entering the pore vestibule but escaping before fully threading through the constriction. (TIF) [file pone.0025723.s001.tif]

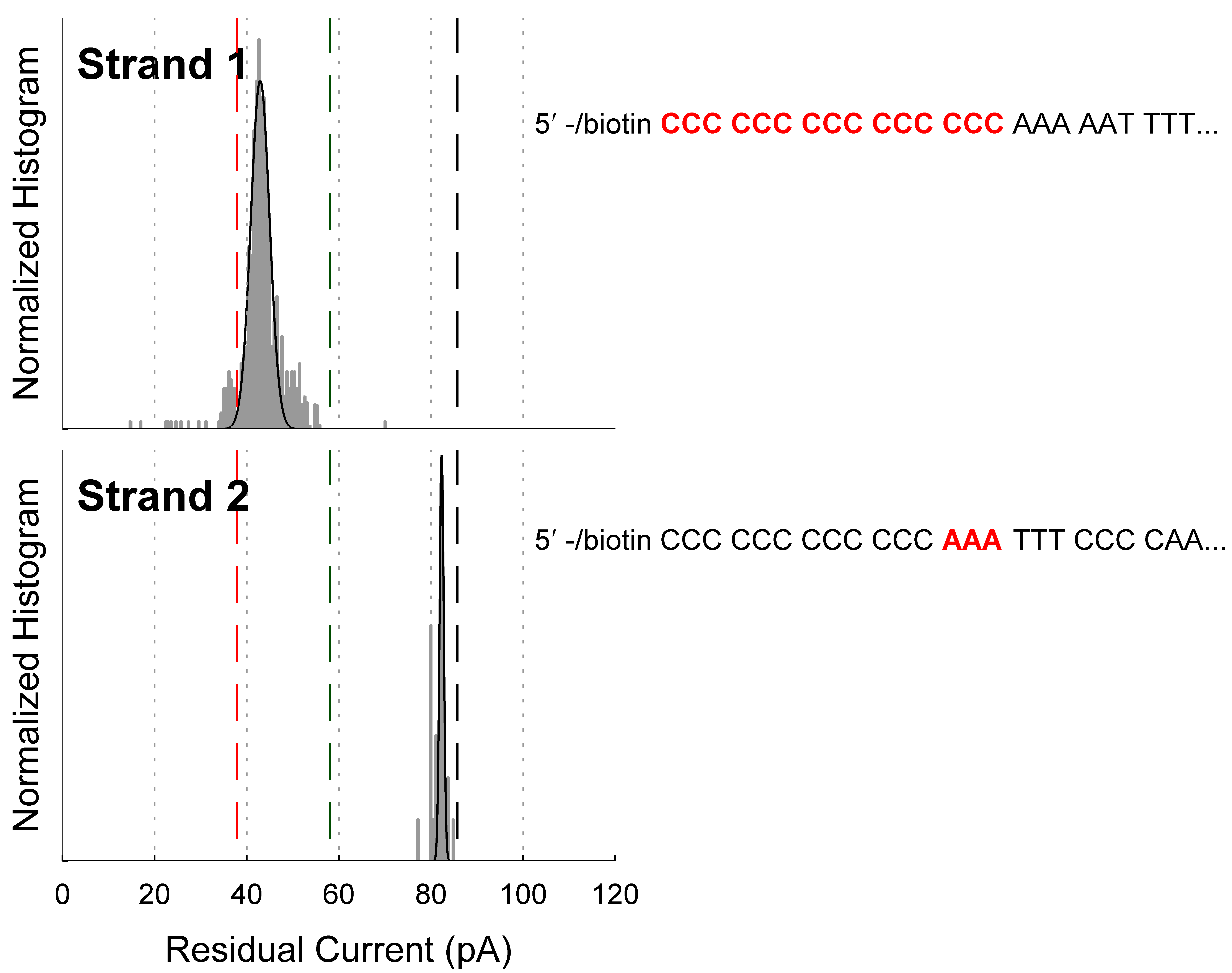

Supplement: Figure S2 — Region of sensitivity. To determine the region of sensitivity of M1-MspA, the residual ion current was measured when the two strands of ssDNA shown below were held within the pore with a NeutrAvidin ‘anchor’. The mean residual ionic current (gray) and fitted Gaussian curve (black) are shown for each strand. The mean residual ionic current for ‘Strand 1’ is most like that of poly-dC, suggesting a recognition site within the first 15 bases (red). The mean residual ionic current for ‘Strand 2’ is most like that of poly-dA, corresponding to a region of sensitivity near the 13th–15th nucleotide (red). For reference, the Gaussian means of the mean residual ionic current are shown for poly-dC (red dashed), poly-dT (green dashed), and poly-dA (black dashed). (TIF) [file pone.0025723.s002.tif]

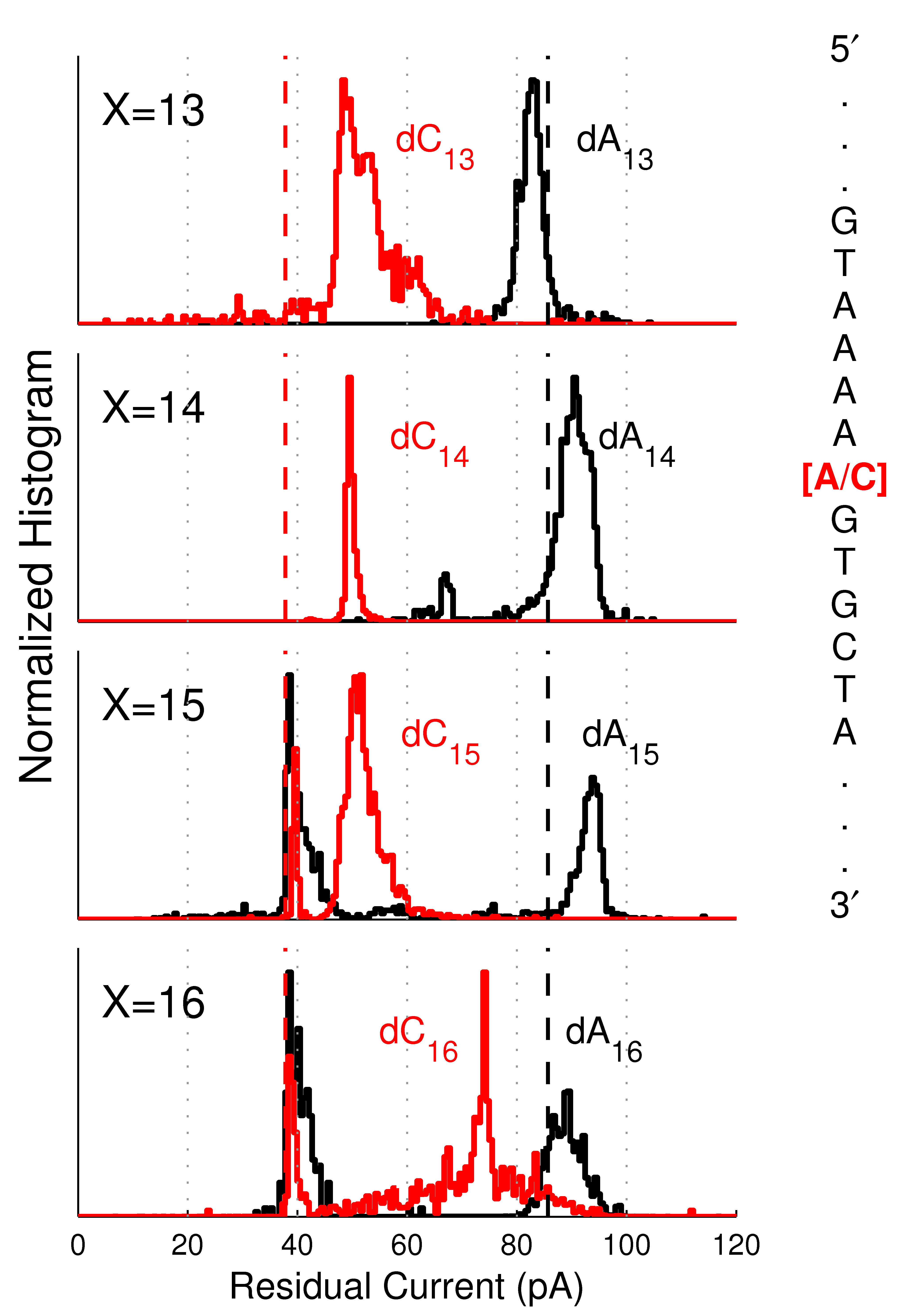

Supplement: Figure S3 — SNP histograms (rs1447295). A segment of ssDNA containing SNP rs1447295, which is associated with an increased risk of prostate cancer [25], [26], was bound to NeutrAvidin such that the polymorphism is at the 13–16th nucleotide, X, from the NeutrAvidin. Part of the surrounding sequence is shown with the nucleotide of interest, either an adenine or cytosine, highlighted in red (see Table S1 for complete sequences). Histograms of the mean residual ionic current for both the dAX and dCX variants for X = 13–16 and Gaussian means of the mean residual ionic current for poly-dA (black dashed) and poly-dC (red dashed) are shown. The two variants are most resolved when X = 13 and 14. The ionic current level for dAX is similar to that of poly-dA and the current for dCX is similar to that found for a single dC in poly-dA. When X = 15 or 16 we observe two current levels for each variation; one level is near 40 pA for both dAX and dCX and the other level is unique to the SNP variation. (TIF) [file pone.0025723.s003.tif]
